# The Mouse Hindbrain As a Model for Studying Embryonic Neurogenesis

**DOI:** 10.3791/56793

**Published:** 2018-01-29

**Authors:** Mathew Tata, Christiana Ruhrberg

**Affiliations:** ^1^UCL Institute of Ophthalmology

**Keywords:** Neuroscience, Issue 131, Neurogenesis, neural progenitor, hindbrain, mammalian development, mouse, mitosis, self-renewal, immunofluorescence, wholemount, floating section, cryosection

## Abstract

The mouse embryo forebrain is the most commonly employed system for studying mammalian neurogenesis during development. However, the highly folded forebrain neuroepithelium is not amenable to wholemount analysis to examine organ-wide neurogenesis patterns. Moreover, defining the mechanisms of forebrain neurogenesis is not necessarily predictive of neurogenesis in other parts of the brain; for example, due to the presence of forebrain-specific progenitor subtypes. The mouse hindbrain provides an alternative model for studying embryonic neurogenesis that is amenable to wholemount analysis, as well as tissue sections to observe the spatiotemporal distribution and behavior of neural progenitors. Moreover, it is easily dissected for other downstream applications, such as cell isolation or molecular biology analysis. As the mouse hindbrain can be readily analyzed in the vast number of cell lineage reporter and mutant mouse strains that have become available, it offers a powerful model for studying the cellular and molecular mechanisms of developmental neurogenesis in a mammalian organism. Here, we present a simple and quick method to use the mouse embryo hindbrain for analyzing mammalian neural progenitor cell (NPC) behavior in wholemount preparations and tissue sections.

**Figure:**
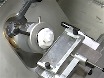


## Introduction

During embryonic mammalian development, NPCs divide in the ventricular (VZ) and subventricular (SVZ) zones of the expanding neuroepithelium to generate new neurons in a process termed 'neurogenesis'. The generation of new neurons and their NPC precursors has been investigated extensively in the forebrain^1^, whilst less is known about this process in other regions.

The forebrain is a complex and intricately folded structure that is largely studied with histological methods after tissue sectioning, which makes understanding neurogenesis patterns across the whole organ challenging. In addition, studies of forebrain neurogenesis are not necessarily predictive of neurogenic behavior in other brain regions or in the spinal cord. For example, signaling cues may elicit differing responses across distinct CNS regions, as observed in the case of ciliary neurotrophic factor and leukemia inhibitory factor, which promote self-renewal of NPCs in the lateral ganglionic eminence, but drive differentiation of spinal cord progenitors^2^. Furthermore, a sub-class of NPCs that populate the developing forebrain and contribute significantly to expansion of the cerebral cortex^1^ are absent from the hindbrain and spinal cord^3^. Inversely, it is conceivable that the spinal cord and hindbrain contain alternative NPC subtypes not present in the cortex.

The mouse embryo hindbrain is the evolutionary oldest region of the mammalian brain and generates the cerebellum and brainstem. In spite of its conservation across species, relatively little is known about hindbrain neurogenesis, including NPC subtypes or their regulation. The majority of hindbrain research in the mouse has focused on the process of tissue segmentation, driven by Hox genes^4^, and the patterning of post-mitotic neurons^5^. In addition, the hindbrain has been used as a model for studying the mechanisms of developmental angiogenesis^6^.

In contrast to the mouse hindbrain, the zebrafish hindbrain has been used extensively to follow NPC differentiation and lineage progression in a vertebrate model organism (*e.g.*,^7^,^8^). The chick hindbrain has also been employed to study neurogenesis during vertebrate development (*e.g.*,^9^,^10^). Similar to the zebrafish hindbrain^11^, the chick hindbrain can be live imaged to study NPC behavior and regulation over time^12^. Analogous longitudinal observation by live imaging is not presently possible in mammalian organisms, because they develop *in utero.* Moreover, targeted manipulation through techniques such as electroporation can be readily applied to free-living zebrafish embryos or chick embryos *in ovo* (*e.g.*,^13^), but such techniques are also more challenging *in utero*.

Nevertheless, the mouse embryo hindbrain is exquisitely suited to define the molecular and cellular mechanisms that govern neurogenesis. Firstly, analysis of the mouse hindbrain will, in many instances, provide information that is more relevant to humans developments than that obtained through studying lower vertebrates. Moreover, a vast number of genetically modified mouse strains are available that can either be used for fate mapping murine NPCs or to alter regulatory mechanisms with constitutive or conditional mutant alleles of relevant genes. Finally, it has recently been shown that microinjection of ventricular hindbrain progenitors *ex vivo* allows at least a brief examination of hindbrain NPC kinetics^14^. Yet, at present, very little is known about the spatiotemporal organization and behavior of hindbrain NPCs in a whole organ context.

Here, we demonstrate a simple and quick method to use the hindbrain as a powerful model for analyzing mammalian NPC behavior in wholemount preparations and tissue sections. We further provide protocols to use immunolabeling for studying different neurogenesis parameters and to process hindbrain samples further for downstream molecular applications such as quantitative reverse transcriptase (qRT)-PCR.

## Protocol

All animal work was carried out in accordance with UK Home Office and local ethical guidelines.

### 1. Summary of Steps and Timing

1. Perform timed matings of adult mice from a strain suitable to answer the biological question under investigation to obtain embryonic day (e) 9.5-e13.5 pregnancies; requires 12 - 15 days.
2. Optionally, prepare 5-bromo-2'-deoxyuridine (BrdU)/5-ethynyl-2'-deoxyuridine (EdU) solution and perform injection (Protocol section 2); requires ~ 1 h on the day before or on the day of embryo isolation, depending on the desired length of EdU/BrdU labeling.
3. Perform embryo isolation and hindbrain dissection (Protocol section 3); requires ~ 10 min/embryo.
4. Perform wholemount immunofluorescence labeling (Protocol section 4); requires 3 days.
5. Section using a vibratome and perform floating section immunofluorescence labeling (Protocol section 4): requires 2 days.
6. Section using a cryostat and perform immunofluorescence labeling of cryosections (Protocol section 5); requires 2 days.

### 2. Inject Pregnant Female Mouse with BrdU or EdU (Optional)

1. Dissolve BrdU or EdU in sterile phosphate buffered saline (PBS) to concentrations of 10 mg/mL and 1 mg/mL, respectively. Caution: BrdU and EdU are toxic; wear appropriate protection.
2. Weigh the pregnant mouse and calculate the volume of BrdU or EdU solution that should be administered to reach 100 mg/kg BrdU or 5 mg/kg EdU.
3. Inject BrdU or EdU solution through the intraperitoneal route either 1 h or 1 day before collecting the embryos, depending on the required length of labeling. NOTE: Labeling for 1 h visualizes hindbrain cells in S-phase. Labeling for 1 day visualizes the progeny of hindbrain NPCs.

### 3. Dissection of Hindbrains from e9.5 - e13.5 Mouse Embryos

1. Euthanize a timed-pregnant female mouse using an ethically approved procedure at the required gestational stage (*e.g.*, cervical dislocation). Using sharp scissors, cut open the peritoneal cavity and carefully excise the uterus. Place the excised uterus containing the embryos into a 60 mm plastic dish containing 20 mL ice-cold PBS. NOTE: Aseptic technique is not required.
2. Perform all further dissection using a dissecting microscope. Using watchmaker forceps number 5, tear the uterine muscle wall to expose the embryos, release each embryo by severing the umbilical cord and remove the yolk sac.
3. Using a sterile Pasteur pipette with a wide-bore opening, transfer each embryo into a clean plastic dish with ice-cold PBS.
4. Using watchmaker forceps number 55, sever the head (Figure 1E).
5. Optionally, retain a small piece of tissue (*e.g.*, ¼ of a yolk sac or approximately 2 mm tail tip) for genomic DNA isolation and subsequent genotyping.
6. Using watchmaker forceps number 55, excise the tissue containing the hindbrain parallel to and immediately below the hindbrain neuroepithelium to separate it from facial and forebrain tissue (Figure 1F).
7. Position the hindbrain and caudal head tissue dorsal side up and identify the 4^th^ ventricle, which is covered by a thin tissue layer (roof plate). Carefully pierce the roof plate using watchmaker forceps number 55 (Figure 1G) and peel away excess tissue with forceps, moving rostrally along the midline over the midbrain, and then caudally over the posterior hindbrain and spinal cord (Figure 1G); the hindbrain should now be exposed in an open book preparation (Figure 1H).
8. Prepare hindbrains for wholemount immunolabeling (Option 1).
  1. Using watchmaker forceps number 55, carefully tease away remaining head mesenchyme and any meninges attached to the pial side of the hindbrain using forceps (Figure 1I). Remove the midbrain and spinal cord tissue (Figure 1J) to leave only the hindbrain tissue (Figure 1K). NOTE: This step of the procedure should not be followed at e9.5 or e10.5, because attempting to do so would likely damage the hindbrain neuroepithelium; moreover, removing the meninges is not essential because the hindbrain neuroepithelium is sufficiently thin at this stage to allow efficient penetration of antibodies for immunolabeling. This step should, however, be followed from e11.5 onwards, when the meningeal tissue consolidates, to enhance antibody penetration into the hindbrain neuroepithelium. Dissection is easiest when performed in ice-cold PBS (use clean PBS for each hindbrain).
9. Prepare hindbrains for vibratome and cryosectioning (Option 2)
  1. Using watchmaker forceps number 55, remove the midbrain and spinal cord tissue (Figure 1J). NOTE: It is not necessary to remove mesenchyme and meninges of hindbrains intended for sectioning (as shown in Figure 1I).
10. Transfer hindbrains from the plastic dish to round-bottomed 2 mL tubes using a Pasteur pipette, aspirate all PBS, and fix for 2 h at 4°C with gentle-agitation in freshly thawed 4% w/v formaldehyde dissolved in PBS. Caution: Formaldehyde is toxic; wear appropriate protection.
11. Rinse hindbrains 3x times with PBS. Store at 4 °C in PBS if immunolabeling will begin within 2-3 days or replace PBS with 50% methanol/50% PBS for 5 min before transferring to 100% methanol for longer storage at -20^°C.^

### 4. Wholemount Immunofluorescence

1. If required, rehydrate hindbrains in serially decreasing dilutions of methanol in PBS (*e.g.*, 75% methanol, 50% methanol, 25% methanol) at room temperature (RT) for 5 min each and then transfer to PBS. NOTE: A graded series of methanol is required to gently rehydrate hindbrains and ensure proper tissue preservation.
2. Permeabilize hindbrains for 30 min at 4 °C in PBS containing 0.1% Triton X-100 (PBT) with gentle agitation.
3. Incubate hindbrains for 1 h at 4 °C in PBT containing 10% heat-inactivated goat serum with gentle agitation. NOTE: Use serum from the host species that the secondary antibodies were raised in. For primary antibodies raised in goat, incubate in serum-free protein block, for example 5% bovine serum albumin in PBS or a suitable commercial alternative (see **Table of Materials**). This will reduce non-specific staining frequently observed when using primary antibodies raised in goat.
4. Incubate hindbrains overnight at 4 °C in PBT containing 1% heat-inactivated serum and primary antibodies with gentle agitation (*e.g.*, rabbit anti-phospho histone H3 [pHH3] diluted 1:400). NOTE: For primary antibodies raised in goat, use PBT without serum.
5. Wash the hindbrains at 4 °C 5x with PBT for 1 h each.
6. Incubate hindbrains overnight at 4 °C in PBT containing appropriate fluorophore-conjugated secondary antibodies (*e.g.*, goat anti-rabbit Alexa Fluor 488) at 1:200 in PBT targeted against the primary antibodies used. Keep hindbrains in the dark from here on to protect fluorophores from photobleaching. NOTE: For primary antibodies raised in goat, use anti-goat Fab fragments of secondary antibodies to reduce non-specific staining.
7. Wash the hindbrains at 4 °C 5x with PBT for 1 h each.
8. Postfix the hindbrains in 4% formaldehyde in PBS for 15 min at RT for long term preservation of antibody binding. Briefly rinse twice in PBS.
9. Cover a glass microscope slide with two layers of black electrical tape and excise a small square from the layered tape to create a pocket large enough to hold one hindbrain.
10. Transfer each hindbrain into a pocket with a Pasteur pipette, remove excess liquid and add an appropriate antifade reagent to the pocket before covering it slowly with a glass coverslip to avoid trapping air bubbles under the coverslip. Seal the coverslip and affix it to the slide with a thin layer of nail polish. Store slide at 4 °C in the dark until image acquisition (Protocol section 7).

### 5. Vibratome Sectioning and Floating Section Immunofluorescence

1. If required, rehydrate hindbrains from methanol as described in step 4.1.
2. Embed hindbrains in molten 3% w/v agarose prepared in distilled water. NOTE: Allow molten agarose to cool to approximately 55°C briefly before embedding to prevent heat damage to hindbrain.
3. Cut transverse hindbrain sections to a thickness of 70 µm using a vibratome. Transfer each freshly cut section with a paintbrush into one well of a 24-well plate containing ice-cold PBS.
4. Label the floating sections as described in steps 4.2-4.7, but modify the steps as follows: wash the floating sections at 4 °C 3x with PBT for 15 min each, decrease incubation time for secondary antibodies to 2 h, and incubate in secondary antibody at RT.
5. Optionally, after labeling with antibodies for other epitopes as described in step 5.4, detect EdU^+^ nuclei using an EdU labeling kit according to the manufacturer's instructions. Incubate the floating sections in the reaction cocktail for 30 min at 37 °C in the dark. Wash hindbrain sections at 4 °C 3x with PBT for 15 min each.
6. Optionally, after labeling for other epitopes as described in step 5.4, detect BrdU^+^ nuclei as follows.
  1. Incubate floating sections in 2 N hydrochloric acid at 37 °C for 30 min in the dark.
  2. Incubate floating sections in 0.1 M sodium borate buffer pH 8.5 twice for 5 min each at RT in the dark to neutralize the hydrochloric acid.
  3. Wash floating sections 3x briefly in PBS at 4 °C.
  4. Perform immunofluorescence labeling for BrdU as described in step 5.4.
7. Incubate the floating sections for 2 min at RT in 10 µg/mL 4',6-diamidino-2-phenylindole dihydrochloride (DAPI) in PBS to counterstain cell nuclei.
8. Postfix the floating sections in 4% formaldehyde for 15 min at RT.
9. Wash floating sections briefly in PBS. Carefully transfer floating sections to a glass microscope slide using a paintbrush. Mop up excess PBS around the section using blotting paper or tissue.
10. Mount sections using 80% glycerol in PBS and cover slowly with a glass coverslip to avoid trapping air bubbles. Store slide at 4 °C in the dark until image acquisition (step 7).

### 6. Cryostat Sectioning and Cryosection Immunofluorescence Labeling

1. If required, rehydrate hindbrains from methanol as described in step 4.1.
2. Incubate hindbrains in 30% w/v sucrose in PBS to cryoprotect hindbrains prior to freezing. NOTE: Hindbrains are ready for freezing when they have sunk to the bottom of the tube, which typically takes ≤ 2 h for e9.5-10.5 hindbrains and 3-6 h for e11.5-13.5 hindbrains.
3. Submerge hindbrains in optical cutting temperature compound (OCT) and freeze quickly by transferring molds containing hindbrains in OCT to isopentane cooled to between -40 °C and -50 °C on dry ice. NOTE: Frozen, embedded hindbrains can be stored at -20 °C short term and -80°C long term until sectioning.
4. Cut transverse hindbrain sections to a thickness of 10 µm using a cryostat and transfer to electrostatically adhesive microscope slides. NOTE: Hindbrain cryosections can be stored at -20 °C short term and -80 °C long term until labeling.
5. Incubate slides at RT for 15 min to dry sections to the slides. Wash cryosections with PBS to dissolve OCT and mark a hydrophobic barrier around cryosections using a PAP pen. Repeat steps 5.4-5.8.
6. Repeat step 5.10 using a polyvinyl alcohol-based mounting medium in place of glycerol.

### 7. Image Acquisition

1. Image samples using an epifluorescent or confocal laser-scanning microscope equipped with lenses suitable for aqueous media-mounted slides and optical filters suitable for the fluorophores used for immunolabeling.
2. To image the whole hindbrain or a hindbrain section, use a lens for 10x magnification (*e.g.*, Figure 2B); to visualize hindbrains areas for quantification, use a 40X magnification (*e.g.*, Figure 2D) and to visualize individual cells, use a lens with a 63x magnification (*e.g.*, Figure 2C).

### 8. Alternative Methods

1. Following step 3.8, homogenize unfixed hindbrains to produce a single cell suspension for **flow cytometry** applications^15^.
2. Following step 3.8, homogenize unfixed hindbrains to extract RNA from single cell suspensions for **RT-qPCR** (*e.g.*, 16). NOTE: Ensure all reagents and equipment are kept sterile and RNase-free throughout to prevent degradation of RNA.
3. Following step 3.8, homogenize unfixed hindbrains to isolate NPCs and propagate them *in vitro* as **neurospheres** for analysis of hindbrain NPC behavior^17^. NOTE: Ensure all reagents and equipment are kept sterile to prevent bacterial/fungal contamination of neurosphere cultures.

## Representative Results

This section illustrates examples of results that can be obtained when studying neurogenesis in the mouse embryonic hindbrain through wholemount and tissue section analysis.

We show that wholemount immunolabeling of the microdissected hindbrain with an antibody for the mitotic marker pHH3 visualizes dividing NPCs in the VZ (Figure 2B **- D**). We show pHH3^+^ NPCs at a high magnification to highlight different stages of mitosis (Figure 2C). We have illustrated that this labeling method is suitable to be performed across several consecutive stages of hindbrain development to observe the time course of NPC mitosis in this organ (Figure 2D).

We show that imaging transverse immunolabeled vibratome sections of the hindbrain 1 h after EdU injection, visualizes the cleavage orientation of mitotic NPCs (Figure 3B), the pseudostratified, interkinetic nuclear migration pattern of cycling progenitors^18^ (Figure 3B, **D**), and the overall VZ structure (Figure 3B** - D**). Note that mitotic pHH3^+^ NPCs are present only at the ventricular surface and not more basally (Figure 3C), which contrasts the basal division pattern of more committed NPCs in the forebrain^19^.

We also illustrate how cycling NPCs and their differentiated progeny can be labeled with BrdU or EdU to assess NPC lineage progression (Figure 4). The immunolabeling of transverse cryosections of the mouse hindbrain 1 day after BrdU injection for BrdU and Ki67demonstrates the number and positioning of cycling NPCs in the neuroepithelium (Figure 4A,** B**), whereby the number of self-renewing NPCs can be defined by calculating the percentage of Ki67^+^ BrdU^+^ cells amongst all BrdU^+^ cells (Figure 4A, **C**).

Finally, we show an example of immunolabeling of hindbrain vibratome sections for RC2, an antigen in the neural-specific intermediate filament nestin, to visualize NPC endfeet (Figure 5B) and processes (Figure 5C). Vibratome sections, rather than thin cryosections, allow improved observation of the highly branched NPCs and also of overall neuroepithelial structure.

**Figure:**
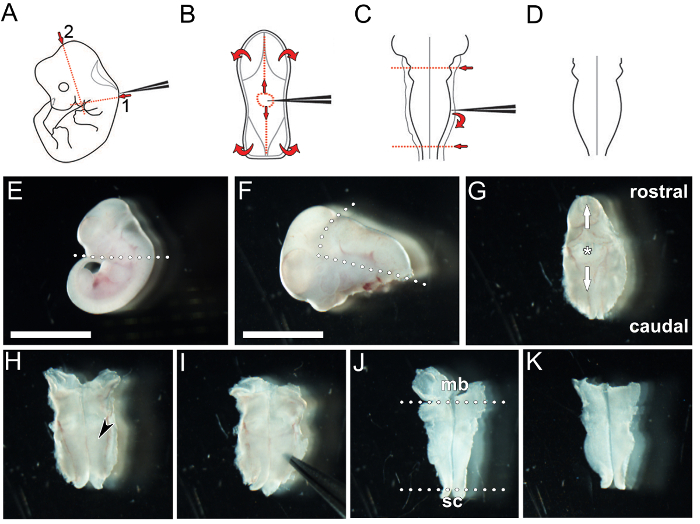

Figure 1**: Key steps in the dissection of a hindbrain from an e11.5 mouse embryo.** (**A - D**) Schematic depiction of hindbrain microdissection. (**A**) The caudal head tissue is removed by severing along the red dotted lines. (**B**) The roof plate is pierced and then torn away in both rostral and caudal directions along the midline (vertical red lines) and then laterally to reveal the hindbrain neuroepithelium. (**C**) The neuroepithelium is pried away from the meninges after inserting the forceps between both structures, and the midbrain and spinal cord tissues are removed by severing the tissue with the forceps along the red dotted lines. (**D**) This procedure yields a flattened hindbrain. (**E-K**) Image capture of key stages in the hindbrain microdissection procedure. (**E**) The embryo is decapitated by severing along the dotted line. (**F**) The hindbrain and 4^th^ ventricle, enclosed by the roof plate, are excised and separated from the head by severing along the dotted lines. (**G**) The 4^th^ ventricle is orientated upwards before a small hole is pierced into the roof plate (asterisk). The hole is widened by peeling the tissue carefully both rostrally and caudally along the midline (arrows) to expose the hindbrain. The hindbrain is positioned pial side down and left to fold out into an open book preparation (**H**; black arrowhead indicates hindbrain neural tissue). If the hindbrain is required for wholemount immunolabeling, the pial membrane is removed by gently prying the hindbrain neuroepithelium from the surrounding meningeal membranes using forceps (**I**). Excess midbrain (mb) and spinal cord (sc) tissue is removed (dotted lines in **J**) to isolate the hindbrain (**K**). Scale bar: 500 µm for (**E**), 300 µm for (**F - K**). Please click here to view a larger version of this figure.

**Figure:**
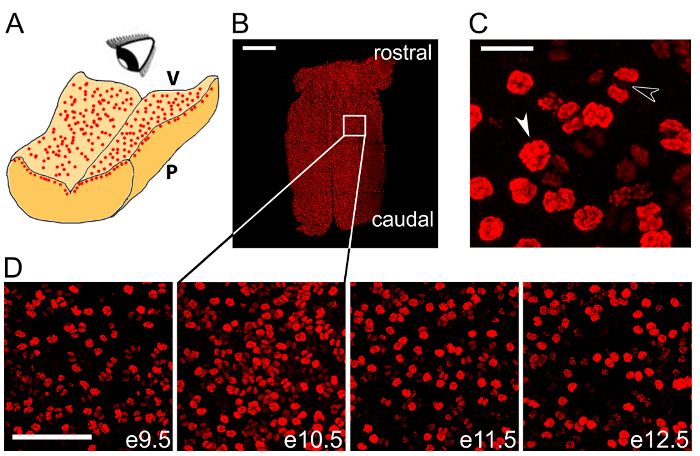

Figure 2**: Wholemount immunolabeling can be used to quantify NPC mitoses across the hindbrain.** (**A**) Schematic illustrating *en face* imaging of mitotic NPCs in the hindbrain ventricular layer. V, ventricular hindbrain side; P, pial hindbrain side. (**B**) Confocal tile scan z stack of e10.5 hindbrain following wholemount immunolabeling for the mitotic marker pHH3 (red). The white box indicates the area shown at higher magnification in the second panel in (**D**). (**C**) Pre-anaphase (white arrowhead) and anaphase (black arrowhead) mitotic figures are distinguishable within the total cohort of mitotic NPCs. (**D**) Time course of wholemount pHH3 labeling from e9.5-13.5. Scale bars: 500 µm in (**B**); 20 µm in (**C**); 100 µm in (**D**). Please click here to view a larger version of this figure.

**Figure:**
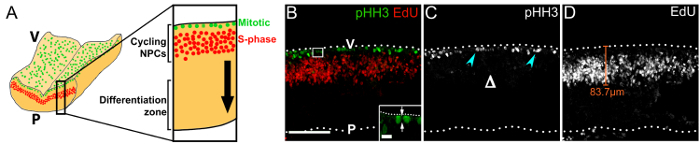

Figure 3**: Cycling NPCs within their germinal zone in the rostral hindbrain.** (**A**) Schematic depicting the position of mitotic (green) and S-phase (red) NPCs at the hindbrain ventricular surface and in the periventricular area, respectively. A virtual cross-section through the hindbrain is displayed at higher magnification on the right. The black arrow indicates the direction of migration of NPC progeny that have exited the cell cycle towards the differentiation zone. (**B**) Confocal z stack of a floating e11.0 hindbrain section from an embryo that received a 1 h EdU pulse; immunolabeling for pHH3 and EdU was used to visualize mitotic (green) and S-phase (red) NPCs, respectively. Hindbrain surfaces are denoted by dotted lines; V, ventricular hindbrain side; P, pial hindbrain side. The area indicated by a white box is shown at higher magnification in the inset; arrows indicate the cleavage plane of an NPC in anaphase, relative to the ventricular surface. (**C**) Single channel displaying pHH3 immunolabeling only. Mitotic NPCs are indicated by cyan arrowheads; the lack of basal divisions is indicated by Δ. (**D**) Single channel displaying EdU labeling only. EdU^+^ NPCs adopt a pseudostratified distribution due to their interkinetic nuclear migration. The distance that NPCs migrate away from the ventricular surface can be measured, as indicated with a representative orange line. Scale bars: 100 µm in (**B - D**); 10 µm in inset in (**B**). Please click here to view a larger version of this figure.

**Figure:**
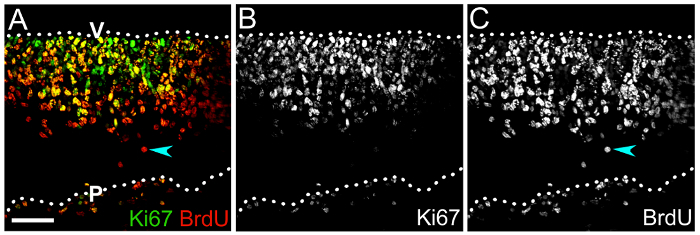

Figure 4**: Combining BrdU with Ki67 labeling to determine hindbrain NPC self-renewal capacity.** (**A**) Confocal z stack of an e10.5 hindbrain cryosection after labeling for Ki67 (green) and BrdU (red) following a 1 day BrdU pulse. Double-positive cells in the ventricular zone are NPCs that have incorporated BrdU and are still moving through the cell cycle, as indicated by Ki67 labeling. The proportion of double-labeled cells amongst the total BrdU^+^ population represents the percentage of self-renewing NPCs. (**B**, **C**) Single channels displaying Ki67 (**B**) and BrdU (**C**) immunolabeling only. A BrdU^+^ cell that lacks Ki67 and therefore has likely exited the cell cycle is indicated by a cyan arrowhead in (**A**,**C**). Hindbrain surfaces are denoted by dotted lines; V, ventricular hindbrain side; P, pial hindbrain side. Scale bar: 50 µm. Please click here to view a larger version of this figure.

**Figure:**
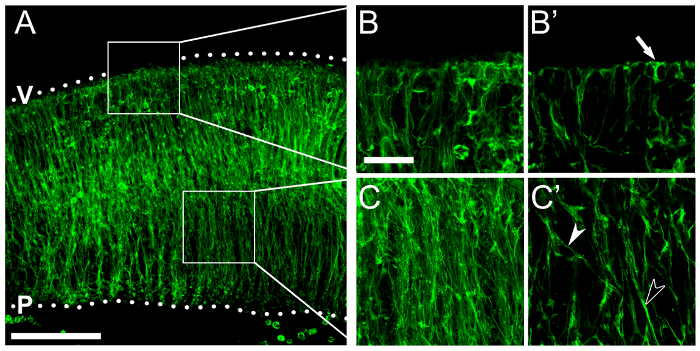

Figure 5**: Nestin immunolabeling visualizes NPC processes and endfeet.** (**A - C**) Immunofluorescence for RC2, which recognizes an epitope on the neural intermediate filament nestin, illustrates NPC morphology across the hindbrain neuroepithelium. (**A**) Confocal z stack of a floating section from an e11.5 hindbrain following RC2 immunolabeling; boxed areas are shown at higher magnification in (**B**, **B'**; apical/ventricular region) and (**C**, **C'**; basal region). Hindbrain surfaces are denoted by dotted lines; V, ventricular hindbrain side; P, pial hindbrain side. (**B**, **C**) Confocal z stacks of boxed areas in (**A**); single optical sections are shown in (**B'**, **C'**), respectively. An NPC apical endfoot anchored at the ventricular surface is indicated by an arrow in (**B'**). Single and fasciculated NPC processes are indicated by white and black arrowheads in (**C'**), respectively. Scale bars: 100 µm, (**A**); 25 µm for (**B, C**). Please click here to view a larger version of this figure.

## Discussion

This protocol describes how to use the mouse embryonic hindbrain as a model to study the mechanisms of developmental neurogenesis. Using a variety of different immunolabeling methods, hindbrain NPCs can be visualized and their number quantified in tissue sections or across organ wholemounts. The ease of dissection and flat anatomy ensures that the hindbrain can be imaged in an 'open book' preparation to gather information on organ-wide neurogenesis patterns.

We further show that NPC morphology and cell cycle-related NPC positioning can be easily visualized in floating- or cryosections of the hindbrain. Both behaviors may be exploited to define new progenitor populations, as has previously been performed in the mammalian telencephalon^20^. For example, early-formed *Sox2*^+^ neuroepithelia and *Pax6*^+^ apical radial glia are present in the hindbrain^21^,^22^, but the hindbrain lacks *Tbr2*^+^ basal progenitors^3^.

The protocol described here can also be adapted to observe the behavior of specific NPC subpopulations by fluorescent labeling for live imaging and/or lineage tracing in fixed tissues. This can be achieved, for example, by studying hindbrains from mice carrying the tamoxifen-inducible *Sox1-iCreERT2* transgene and the *Rosa26^tdTomato^* reporter^23^.

In addition to enhancing knowledge of murine neurogenesis, studying the hindbrain may elucidate broadly relevant neurogenic mechanisms that are shared across species, because the hindbrain is a highly conserved brain region that is expected to be more similar between vertebrate species than the forebrain.

As hindbrain neurogenesis takes place over a comparatively shorter time window than forebrain neurogenesis^23^, it is important to consider the need for comparing adequately staged embryos. Accordingly, experimental bias is avoided by counting and recording the number of somite pairs in an embryo prior to isolating its hindbrain. The hindbrain tissue itself is fragile and forceps should therefore be handled carefully when separating the hindbrain tissue from the head mesenchyme and meninges; a couple of 'practice runs' might therefore be advisable before dissection of valuable embryos is attempted. Furthermore, hindbrains should be transferred from one tube to another using a wide-bore Pasteur pipette rather than with forceps to avoid damage (the pipette's opening can be widened by cutting of the tip with clean scissors). Finally, although uncommon, the extent of EdU/BrdU incorporation into S-phase NPCs may be variable, in particular during short (*i.e.*, 1 h) pulses. To improve labeling, ensure that EdU/BrdU is dissolved properly before loading the solution into the syringe and inject carefully into the peritoneal cavity. Poor injections, such as those made subcutaneously by accident, will result in losing or trapping the EdU/BrdU solution and prevent it from entering the circulation.

## Disclosures

None of the authors have competing interests or conflicting interests.
